# Exposure of domestic animals to *Mayaro* and *Oropouche viruses* in urban and peri-urban areas of West-Central Brazil

**DOI:** 10.1186/s42522-024-00104-w

**Published:** 2024-07-01

**Authors:** Helver Gonçalves Dias, Débora Familiar-Macedo, Ingrid Oliveira Garrido, Flávia Barreto dos Santos, Alex Pauvolid-Corrêa

**Affiliations:** 1 Laboratório das Interações Vírus-Hospedeiros, Instituto Oswaldo Cruz, Fundação Oswaldo Cruz (Fiocruz), Rio de Janeiro, RJ 21040-900 Brazil; 2 Laboratório de Virologia Veterinária de Viçosa, Departamento de Veterinária, Universidade Federal de Viçosa, Viçosa, MG 36570-900 Brazil

**Keywords:** Serosurvey, Arbovirus, Neutralizing antibodies, Domestic animals, One health

## Abstract

*Oropouche* and *Mayaro viruses* are enzootic arboviruses of public health concern throughout Latin America. Recent outbreaks of OROV in northern region and sporadic autochthonous cases in western region of Brazil, suggest a silent circulation of these neglected viruses. Aiming to investigate the exposure of different species of domestic animals to MAYV and OROV in urban and peri-urban areas of West-Central Brazil, we performed a cross-sectional serosurvey by plaque reduction neutralization test (PRNT). Our findings included neutralizing antibodies for both arboviruses in cattle, dogs and horses, suggesting eventual role of domestic animals in enzootic arbovirus surveillance in Brazil.

## Introduction

*Mayaro virus* (MAYV, *Togaviridae*) and *Orthobunyavirus oropoucheense* (OROV, *Peribunyaviridae*) are mosquito-borne enzootic arboviruses involved in outbreaks of human febrile illness, mostly reported in northern South America, including the Amazon region in northern Brazil, and neighboring countries [1, 2]. Although most infections in humans are asymptomatic, symptomatic individuals mostly present mild acute febrile illness in a self-limiting clinical course. Few individuals will present intense and sometimes chronic arthralgia caused by MAYV, and meningitis or meningoencephalitis caused by OROV [3–5].

Recent reports show that both viruses circulate in urban areas of West-Central and Northeast regions of the country [5–9]. During outbreaks, MAYV and OROV are frequently detected in dipterans, such as *Haemagogus janthinomys* mosquitoes and *Culicoides paraensis* midges, respectively. Evidences of MAYV and OROV in urban areas raise concerns among the potential urbanization of their transmission cycles [2, 10].

Because domestic animals are commonly found in urban and peri-urban areas, they can be exposed to arboviruses to the same extent as humans. Even though domestic animals rarely act as amplifying hosts for most arboviruses, they may be important for surveillance, due to their exposure to arthropod vectors and proximity to wildlife and humans [2, 11, 12]. Several studies have demonstrated that some domestic animals, such dogs and horses, may act as useful sentinels for arbovirus’ circulation [12–14]. Previous studies conducted in West-Central Brazil have showed the exposure of domestic animals to several enzootic arboviruses, including MAYV and OROV, in addition to epidemic arboviruses such as Zika virus [12–14]. Here, we aim to assess the presence of neutralizing antibodies (NAb) for MAYV and OROV by plaque reduction neutralization test (PRNT) in plasma samples of several species of domestic animals from urban and peri-urban areas of the states of Mato Grosso and Mato Grosso do Sul, West-Central Brazil.

## The study

**Fig. 1:**
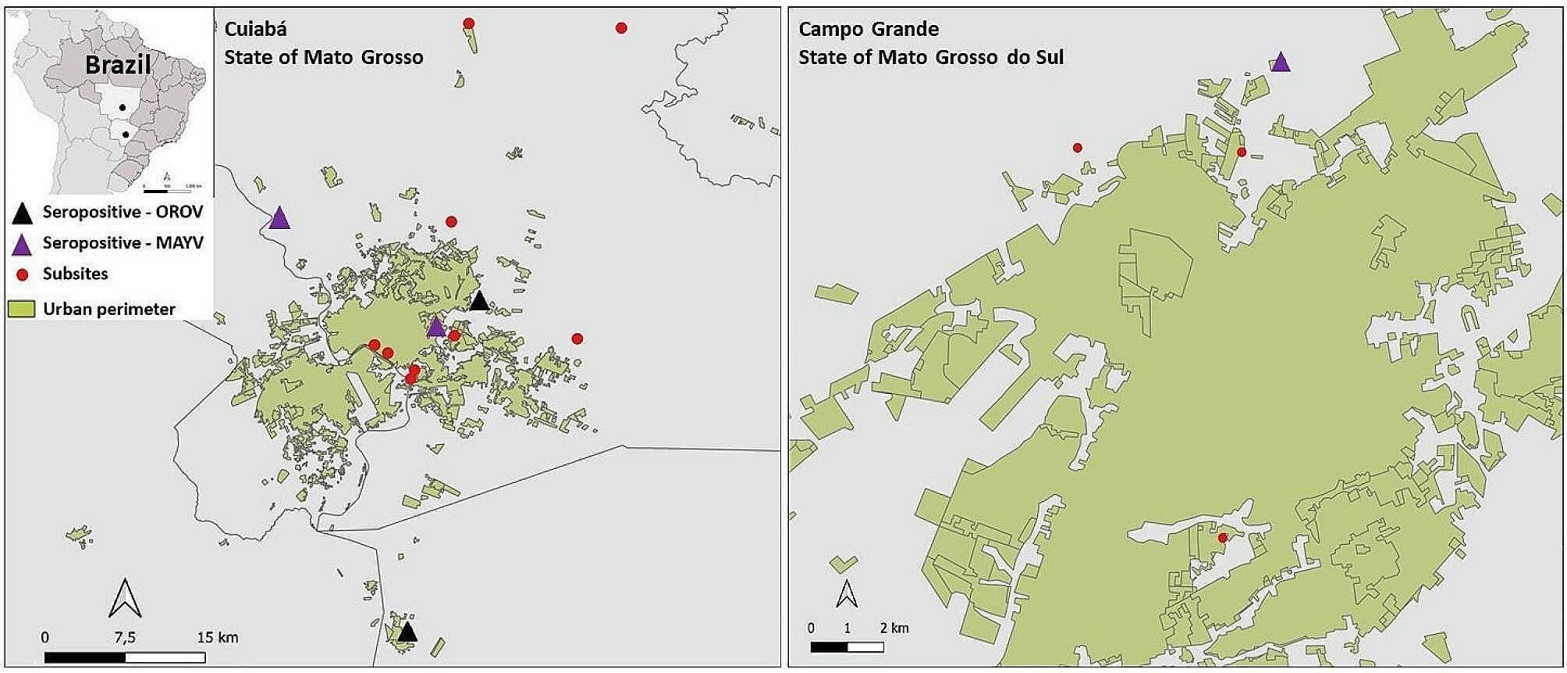
Subsites used for sampling of domestic animals in the West-Central region of Brazil

We performed a cross-sectional serosurvey and convenience sampling in domestic animals from 17 subsites in urban and peri-urban areas of Campo Grande, capital of the state of Mato Grosso do Sul (MS) and Cuiabá, capital of the state of Mato Grosso (MT), located in West-Central Brazil, between April 2017 and March 2018 (Fig. 1). Animal sampling was approved by the Animal Ethics Committees in compliance with the requirements of Brazilian Law 11,794/2008, decree 6899/2009.

A total of 200 animals were sampled, including cattle (*Bos indicus taurus*, *n* = 40; 20%), chickens (*Gallus gallus domesticus*, *n* = 40; 20%), horses (*Equus ferus caballus*, *n* = 35; 17,5%), dogs (*Canis lupus familiaris*, *n* = 30; 15%), sheeps (*Ovis aries*, *n* = 30, 15%), cats (*Felis catus*, *n* = 20, 10%) and pigs (*Sus scrofa domesticus*, *n* = 5, 2,5%). Sampled animals were grouped by species, sex and age group, and sampling sites included private and public shelters, ranches, campuses of local universities, state police equine facilities and equestrian societies, residential neighborhoods, zoonosis control centers and veterinary hospitals.

For detection of NAb for MAYV and OROV by PRNT, plasma samples were heat-inactivated and 2-fold serial diluted from 1:20 to 1:640 with standardized concentrations of MAYV (ATCC VR 66, strain TR 4675, GenBank #MK070492) and OROV (Strain BeAn 19,991, GenBank accession #KP052852.1, #KP052851.1, #KP052850.1). The PRNT was conducted in 6-well plates containing VERO CCL-81 cells. We used a conservative threshold for detection of NAb of 90%, and we considered seropositive only samples that presented PRNT_90_ titers of 20 or greater [12, 13].

The short viremia period associated with arbovirus infection may result in underreporting of exposed animals if only molecular tools are used for diagnosis [12]. It is also worth mentioning that, in a previous study, MAYV and OROV RNA was not detected in these samples [11]. Therefore, serosurveys for the detection of NAb are a reliable approach to confirm enzootic arbovirus exposure during surveillance programs.

Overall, 5.5% of animals tested had NAb for MAYV and/or OROV. Seven animals presented NAb for MAYV, five (71,4%) cattle, one (14,3%) horse and one (14,3%) dog. Six animals had NAb for OROV, three (50%) cattle and three (50%) dogs. One dog and one cow presented NAb for both MAYV and OROV (Table 1).

**Table 1:** Domestic animals from urban areas of West-Central Brazil presenting NAb for MAYV and/or OROV in PRNT_90_ assay

Sample ID	Species	Sampling state	Age group	Sex	Virus	PRNT_90_ titer
AG0346	*Bos indicus/taurus*	MS	-	-	MAYV	20
AG0348	*Bos indicus/taurus*	MS	Subadult	M	MAYV	20
AG0329	*Bos indicus/taurus*	MS	Adult	F	MAYV	80
AU0366	*Equus ferus caballus*	MT	Subadult	M	MAYV	40
AU0275	*Bos indicus/taurus*	MT	Adult	F	MAYV	40
AU0213	*Bos indicus/taurus*	MT	Adult	F	OROV	20
AU0114	*Canis lupus familiaris*	MT	Adult	F	OROV	40
AU0124	*Canis lupus familiaris*	MT	Adult	F	OROV	40
AU0215	*Bos indicus/taurus*	MT	Adult	F	OROV	40
AU0112	*Canis lupus familiaris*	MT	Subadult	M	MAYV/OROV	20/320
AU0424	*Bos indicus/taurus*	MT	Subadult	M	MAYV/OROV	160/160

M = Male; F = Female; - = age not recorded; PRNT^90^: plaque reduction neutralization test with 90% neutralization threshold; MAYV: Mayaro virus; OROV: Oropouche virus; MT = State of Mato Grosso; MS = State of Mato Grosso do Sul; Juvenile = ≤ 6 months-old; Subadult = 6–12 months-old; Adult = > 12 months.

Our findings suggest previous exposure of domestic animals to MAYV and OROV in Cuiaba and Campo Grande, populated cities located in the West-Central Brazil. Although the possibility of cross-reaction cannot be completely ruled out, evidences of MAYV and OROV infections in humans and vectors from the same area corroborate the results presented here [5, 7, 13–15]. Together, these findings reveal that MAYV and OROV have been circulating in populated areas of West-Central Brazil. Moreover, serum samples from humans and backyard chickens collected in 2019 in the hinterland of the state of Ceará, located in Northeast Brazil, showed hemagglutination-inhibiting antibodies to MAYV [16]. OROV detection in human, vectors and animal samples in populated areas is a major concern. Molecular detection of OROV in the states of Bahia and Minas Gerais, touristic and densely populated areas in Brazil, reveals the potential for transmission and dissemination to other areas in the country and elsewhere [5, 6].

Prediction models of risk areas and outbreak modeling indicate that different regions are favorable to the dispersion of OROV and MAYV throughout Brazil and other Latin American countries [1, 10, 17]. These data associated to recent detection of MAYV and OROV outside Amazon in the absence of outbreaks suggests that the circulation of these viruses in Brazil may be underreported in non-enzootic regions.

### Limitations

Limitations of our study included the limited species of alphaviruses and orthobunyaviruses used for the differential diagnosis by PRNT. Even though we only considered as OROV or MAYV-seropositive those samples with a titer greater than 20 and used a conservative threshold of 90% of neutralization, other related viruses may be circulating in our study sites. Sampling bias can be considered as a limitation, since the animals were sampled by convenience.

## Conclusions

The findings presented here suggest that dogs, horses and cattle sampled in populated cities of West-Central Brazil have been exposed to MAYV and OROV. These results raise concern about the potential urbanization of MAYV and OROV in Brazil, and show that comprehensive data on the serological status of domestic animals’ populations can be important for arboviruses’ surveillance that ultimately may guide public policies.

## Data Availability

All the data available is included in the manuscript.

## References

[1] Caicedo EY, Charniga K, Rueda A, Dorigatti I, Mendez Y, Hamlet A (2021). The epidemiology of Mayaro virus in the Americas: a systematic review and key parameter estimates for outbreak modelling. Corrales-Aguilar E, organizador. PLoS Negl Trop Dis 3 de junho de.

[2] Dias HG, dos Santos FB, Pauvolid-Corrêa A (2022). An overview of neglected orthobunyaviruses in Brazil. Viruses 7 de maio de.

[3] Azevedo RSS, Silva EVP, Carvalho VL, Rodrigues SG, Neto JPN, Monteiro HAO (2009). Mayaro Fever Virus, Brazilian Amazon. Emerg Infect Dis novembro de.

[4] Chiang JO, Azevedo RS, Justino MCA, Matos HJ, Cabeça HLS, Silva SP (2021). Neurological disease caused by Oropouche virus in northern Brazil: should it be included in the scope of clinical neurological diseases?. J Neurovirol Agosto de.

[5] Fonseca LM, dos Carvalho S, Bandeira RH, Sardi AC, Campos SI (2020). Oropouche Virus Detection in Febrile patients’ saliva and urine samples in Salvador, Bahia, Brazil. Jpn J Infect Dis.

[6] Nunes MRT, Martins LC, Rodrigues SG, Chiang JO, Azevedo R do S da, da Rosa S APA, editors. Oropouche Virus Isolation, Southeast Brazil. Emerg Infect Dis. outubro de 2005;11(10):1610–3. 10.3201/eid1110.050464 PMC3366749 16318707

[7] Serra OP, Cardoso BF, Ribeiro ALM, dos Santos FAL, Slhessarenko RD (2016). Mayaro virus and dengue virus 1 and 4 natural infection in culicids from Cuiabá, state of Mato Grosso, Brazil. Mem Inst Oswaldo Cruz janeiro de.

[8] De Curcio JS, Salem-Izacc SM, Pereira Neto LM, Nunes EB, Anunciação CE, Silveira-Lacerda EDP (2022). Detection of Mayaro virus in Aedes aegypti mosquitoes circulating in Goiânia-Goiás-Brazil. Microbes Infect junho de.

[9] De Souza Costa MC, Siqueira Maia LM, Costa De Souza V, Gonzaga AM, Correa De Azevedo V, Ramos Martins L (2019). Arbovirus investigation in patients from Mato Grosso during Zika and Chikungunya virus introdution in Brazil, 2015–2016. Acta Trop fevereiro de.

[10] Romero-Alvarez D, Escobar LE, Auguste AJ, Del Valle SY, Manore CA. Transmission risk of Oropouche fever across the Americas. Infect Dis Poverty. 6 de maio de. 2023;12(1):47. 10.1186/s40249-023-01091-2 PMC10163756 37149619

[11] Dias HG, de Lima RC, Barbosa LS, de Souza TMA, Badolato-Correa J, Maia LMS (2022). Retrospective molecular investigation of Mayaro and Oropouche viruses at the human-animal interface in West-central Brazil, 2016–2018. Ikegami T, organizador. PLoS ONE 17 de novembro de.

[12] Pauvolid-Corrêa A, Gonçalves Dias H, Marina Siqueira Maia L, Porfírio G, Oliveira Morgado T, Sabino-Santos G et al. Zika Virus Surveillance at the human–animal interface in West-Central Brazil, 2017–2018. Viruses. 16 de dezembro de 2019;11(12):1164. 10.3390/v11121164 PMC6950091 31888285

[13] Pauvolid-Corrêa A, Campos Z, Soares R, Nogueira RMR, Komar N (2017). Neutralizing antibodies for orthobunyaviruses in Pantanal, Brazil. Aguilar PV, organizador. PLoS Negl Trop Dis 1° De novembro de.

[14] Pauvolid-Corrêa A, Juliano RS, Campos Z, Velez J, Nogueira RMR, Komar N (2015). Neutralising antibodies for Mayaro virus in Pantanal, Brazil. Mem Inst Oswaldo Cruz fevereiro de.

[15] Cardoso BF, Serra OP, Heinen da LB, Zuchi S, Souza N, de Naveca VC (2015). Detection of Oropouche virus segment S in patients and inCulex quinquefasciatus in the state of Mato Grosso, Brazil. Mem Inst Oswaldo Cruz Setembro De.

[16] Löwen Levy Chalhoub F, De Maia E, Holanda Duarte B, Eielson Pinheiro De Sá M, Cerqueira Lima P, De Carneiro A et al. West Nile Virus in the state of Ceará, Northeast Brazil. Microorganisms. 10 de agosto de 2021;9(8):1699. 10.3390/microorganisms9081699 PMC8401605 34442778

[17] Celone M, Pecor DB, Potter A, Richardson A, Dunford J, Pollett S (2022). An ecological niche model to predict the geographic distribution of Haemagogus janthinomys, Dyar, 1921 a yellow fever and Mayaro virus vector, in South America. Lau EH, organizador. PLoS Negl Trop Dis 8 De julho de.

